# A Novel, Inexpensive Portable Respiratory Protection Unit for Prehospital Use: A Technical Note

**DOI:** 10.7759/cureus.7954

**Published:** 2020-05-04

**Authors:** Christopher Sampson, Adam Beckett

**Affiliations:** 1 Emergency Medicine, University of Missouri School of Medicine, Columbia, USA

**Keywords:** ems, covid-19, intubation

## Abstract

There is a heightened concern for exposure to infectious aerosols during the intubation of SARS-CoV-2 (severe acute respiratory syndrome coronavirus 2) patients. We took our previously designed portable, inexpensive, and easily constructed device and modified it for the prehospital setting.

Using polyvinyl chloride pipe and automobile collision wrap, a prehospital unit can be constructed in less than 30 minutes, and the cost of material is under 50 U.S. dollars. The box stores easily in an ambulance and can be assembled on the scene within two minutes.

This easily built device likely provides some limited protection from aerosolization during video laryngoscopy and can be replicated easily.

## Introduction

Some limited experimentation has suggested that aerosolized novel human coronavirus that is named severe acute respiratory syndrome coronavirus 2 (SARS-CoV-2) can remain viable in the air for up to three hours [1]. Simulated studies have shown how health care workers can be at risk from patient secretions [2]. The Centers for Disease Control and Prevention (CDC) has issued guidelines that recommend the use of highest level of personal protective equipment when performing endotracheal intubation, which increases the risk of aerosolization [3]. A limited study has shown a possible benefit of a barrier box providing protection when performing endotracheal intubation [4]. A previously designed inexpensive and easily reproducible intubation protection device for the hospital setting was used [5]. This hospital model was adapted for Emergency Medical Services (EMS) use by modifying its size and making a more portable device that could be easily stored in the ambulance prior to use.

## Technical report

Similar to our previously reported hospital box, the following materials were used to construct the device for EMS use: polyvinyl chloride (PVC) ½-inch pipe, PVC joints, and plastic collision wrap (Table 1).

**Table 1:** Materials required to construct a portable respiratory protection unit

Miter saw or hacksaw
10-feet polyvinyl chloride ½-inch diameter pipe cut up into:
#4 28-inch length
#3 19-inch length
#2 20-inch length
#2 7.5-inch (top bars)
#2 11-inch (bottom bars)
#2 ½-inch “T” fitting
#4 90-degree fittings
#4 3-way fitting
36-inch automobile plastic collision wrap (30-feet roll)

**Figure 1:**
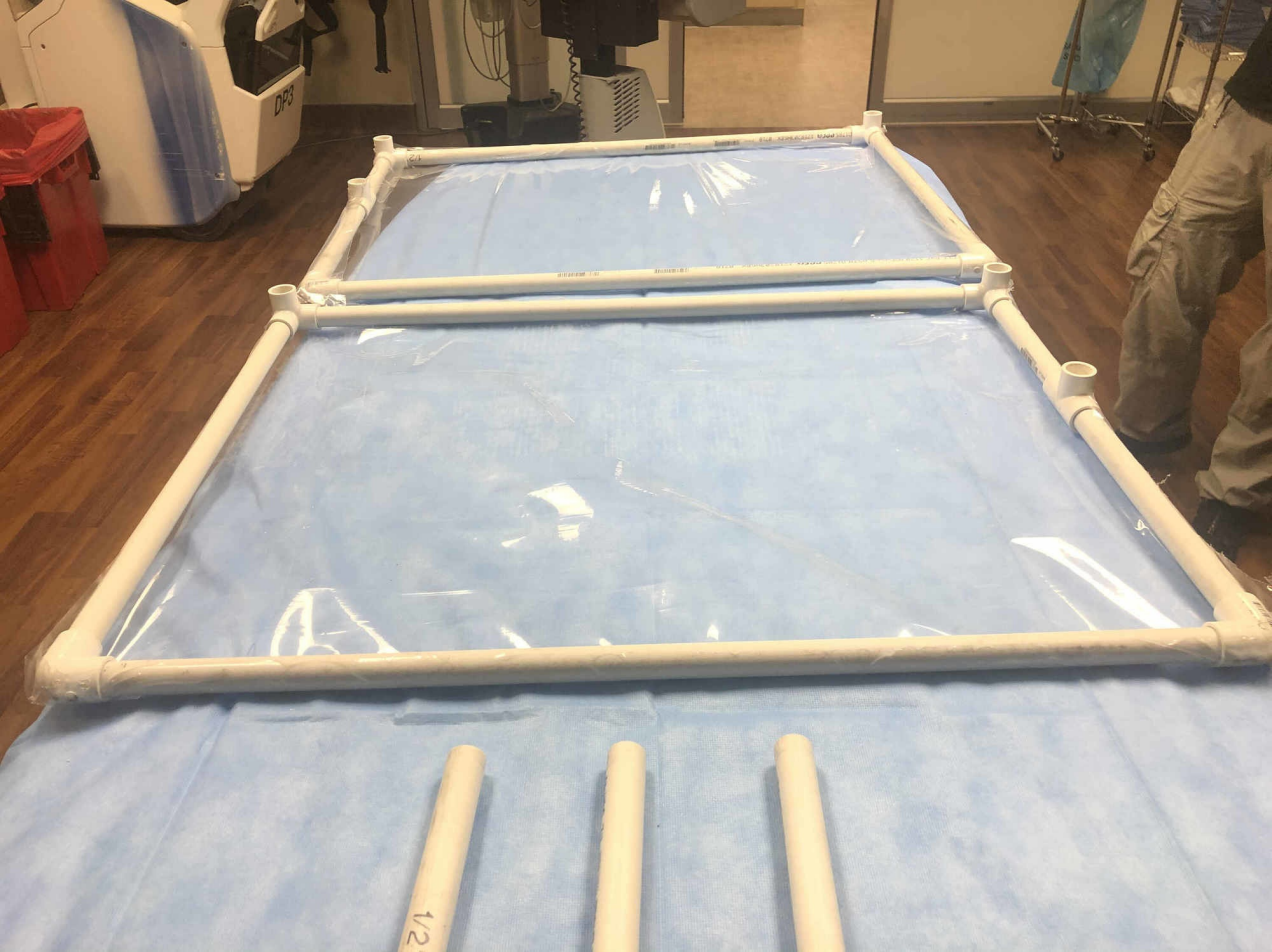
Frame sides

**Figure 2:**
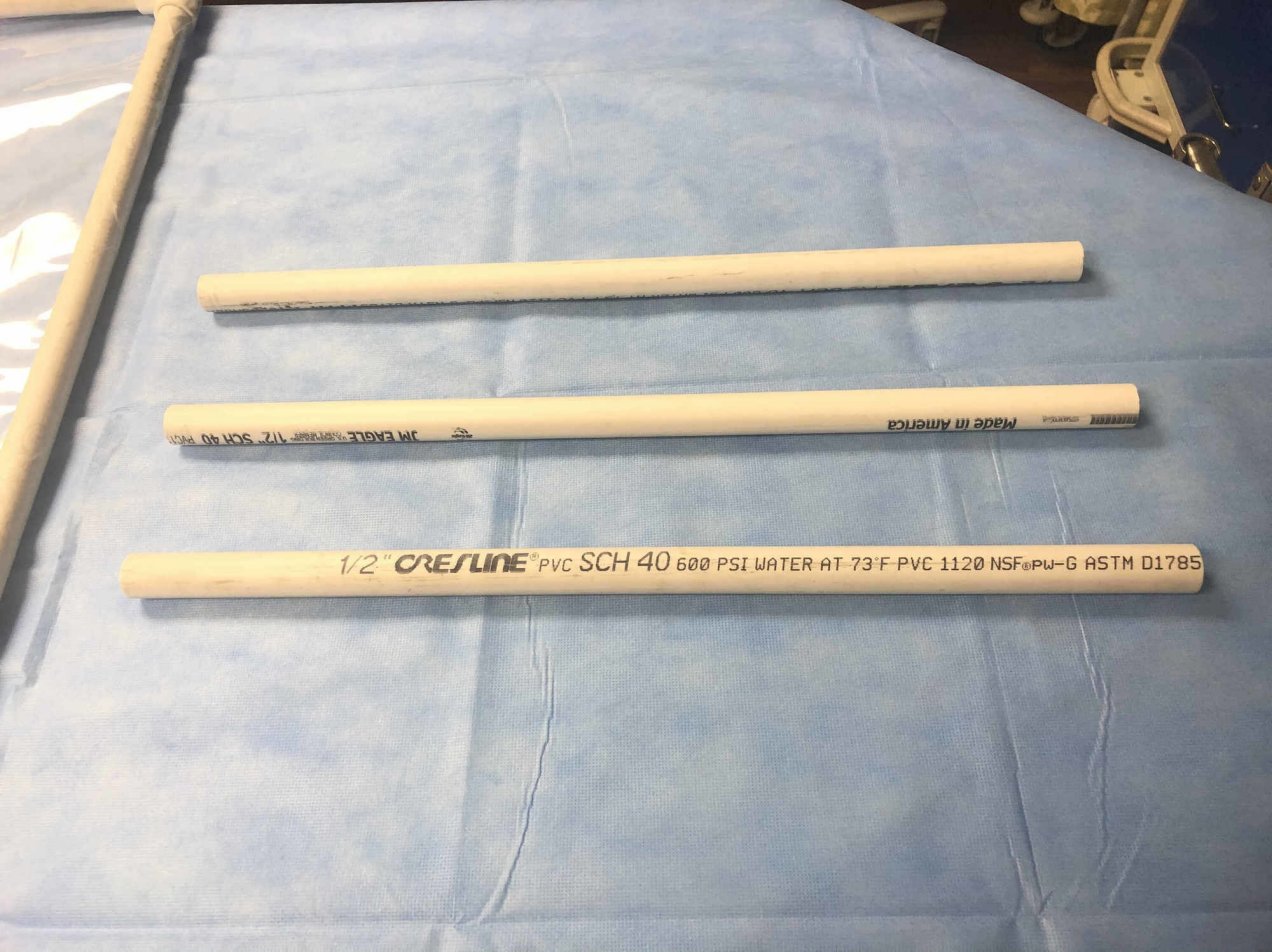
Frame cross-bars

The PVC pipe can be purchased as a single 10-feet piece that can be cut up into thirteen pieces using a miter saw or hacksaw. Completed box dimensions for this device are 24 inches in height, 22 inches wide, and 27 inches in length. We reduced the width in this version from our hospital designed boxes in order to better accommodate the narrower EMS stretcher. For the top four corners, three-way fittings are used, and on the base corners two 90-degree fittings are used. On the posterior (caudal) surface, two "T" fittings are used which will support an additional cross-bar when assembled. Following construction of the frame's two sides, collision wrap is then used to cover each side. Collision wrap has a single adhesive side and is used in automotive industry to temporarily cover broken car windows. The adhesive side of the wrap faces into the interior of the box (Figure 1). The frame cross-bars are not used in the preassembly period (Figure 2).The time it takes to preassemble each EMS box is very quick and can be completed in under 30 minutes with two people working together. Cost of the EMS device remains low, and materials were purchased for less than 50 U.S. dollars. The largest expense was the automobile collision wrap, which can be used multiple times to rewrap the box.

**Figure 3:**
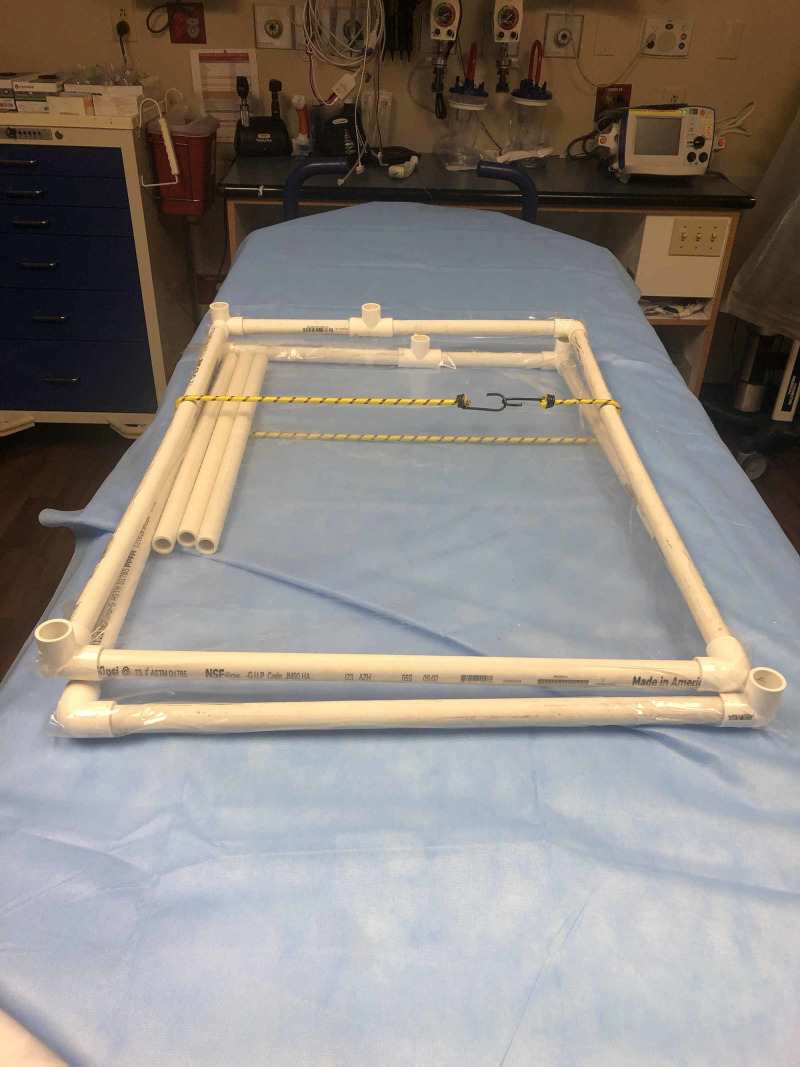
Frame wrapped with a bungee cord for transport

Prior to storage, the two prewrapped sides can be re-stacked on one another. The three cross-bars can then be stored against an adhesive part of the plastic wall, and both side pieces can be strapped together with a bungee cord (Figure 3).

**Figure 4:**
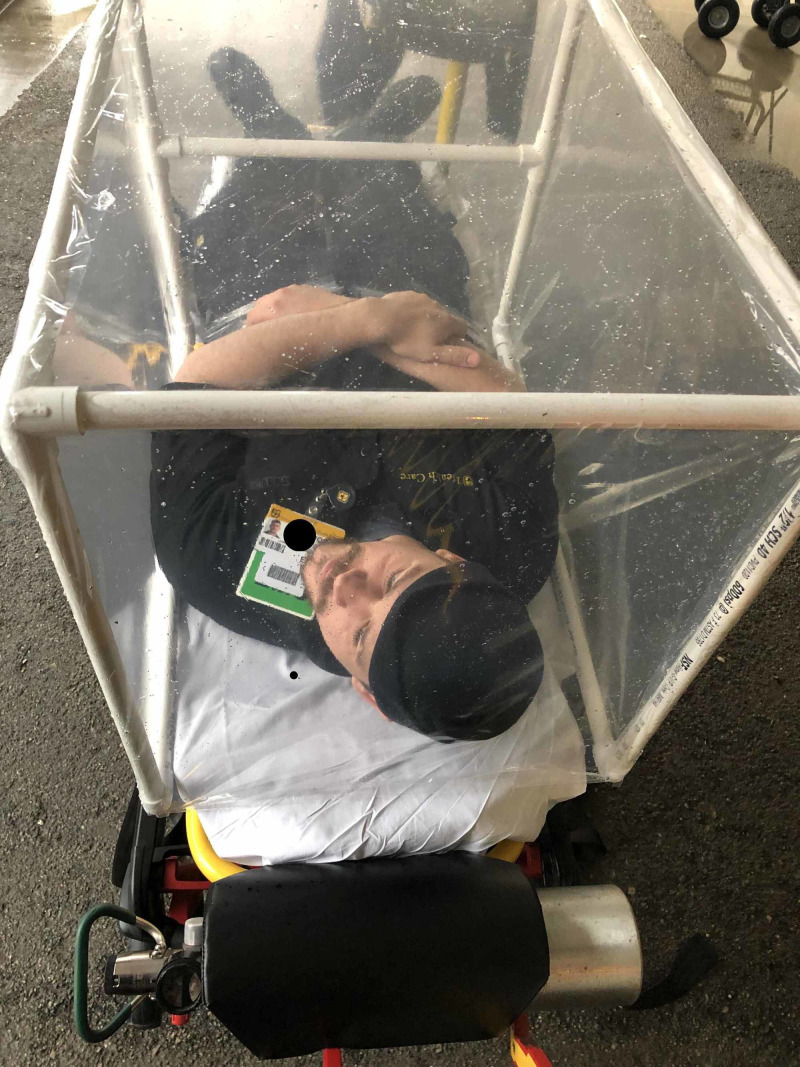
Unit overlying a simulated patient

This compactness enables easy storage in the ambulance. When the box is needed for use on the scene, the parts can be rapidly separated. Prior to intubation, with two people working together, the box can easily be assembled. The three cross-bars are inserted in one side of the frame. The other side of the frame is then attached. Collision wrap can then be used to wrap the box in either a cephalad-to-caudal direction or opposite. Assembly takes approximately two minutes, and the box is now ready for use. During intubation, the box is laid over the patient covering the head and upper chest (Figure 4).

If the box does not fit snugly on the patient and stretcher, a small incision can be made at the junction of the patient's waistline and collision wrap to separate the plastic wrap. Using a knife or any other sharp device, two vertical incisions can be made on the sides of the box to place hands through in order to use a bag valve mask. Additional incisions can be made on the cephalad wall for access to intubate the patient. Equipment can be passed through either the incisions or under the bottom of the plastic covering. The authors recommend the intubation being performed should be assisted by video laryngoscopy. This enables the prehospital clinician to not be required to be near the patient’s oropharynx during the procedure. Following intubation, the box could be removed or even left in place for clinician protection during transport if a disconnection from the endotracheal tube occurred. If left in place for transport, bungee cords could be used to secure the box to the EMS stretcher.

Following use, the plastic can be removed and the frame can be cleaned according to the CDC guidelines with antiviral wipes or sprayed down with an appropriate cleaning agent.

## Discussion

The portable respiratory protection unit (PRPU) can be easily assembled rapidly in the prehospital setting with materials that are readily available in most areas. Given the possible difficulties in acquiring automobile collision wrap, any clear plastic material that could adequately cover the frame could be used. Other suggestions include a clear painter’s drape. Direct visualization of the patient may be limited given the opacity of any alternative material used.

Concern over the adhesive nature of the inner surface and its possible interference with performing airway management has not been experienced in the limited time this box has been used.

Use of the unit during airway intubation is not the only potential use of this device. Other suggestions include using the box as a protective covering for patients during EMS transfer through a hospital in either the awake or intubated COVID-19 (coronavirus disease 2019) patient.

Due to the time-sensitive nature of disseminating this model, a trial of this model in a controlled setting could not be performed. This model does not completely contain aerosolized viral particles, and appropriate personal protective equipment should still be worn when intubating any suspected SARS-CoV-2 patient.

## Conclusions

The prehospital PRPU is an inexpensive, quick, and easy-to-construct protective device for prehospital clinicians to using during intubation of high-risk patients such as those with SARS-CoV-2. The device is easy to reuse and has many additional applications in the prehospital setting.

## Appendices

Instructional video link: https://vimeo.com/403820752
